# Natural Sleep

**Published:** 1892-11

**Authors:** W. H. Maxon


### NATURAL SLEEP.

It would seem absurd indeed to the world in general to say that a large majority of American adults do not know how to sleep, or how to get the full measure of repose that nature has embodied in that function. American people are fast becoming a nervous people. Rapid eating, rapid work, rapid accumulation of means, rapid expenditure, rapid living, and rapid transit are what make up the ever constant whirl of life, until the pulse and the nerves have become enfeebled, and thereby the nerve centres, like a dynamite bomb, are ready to explode on the slightest provocation or concussion. A general anxious feeling or a feeling of unrest hovers over the homes, the cities, and the nation, and while there are many individual exceptions, the larger majority of the people are living in a continual state of expectancy, with nerves alert, and only pacified when excited. Thus the theatre and the play, sensational literature, stimulating foods and beverages, are on the increase. The enormous consumption of nerve medicines and tonics would alone speak loudly to one who studies in the least degree the physical tendencies of medicines. Nothing proves better the artificial state of man in this age than the artificial means by which he tries to adjust himself to nature's laws—means which, in most cases, serve merely to keep up appearances of life a little longer; but the crash comes sooner or later, for any undue stimulation of that which is natural must, in a short time, lead to disease and death.

Over excitement means rapid nerve waste. It is tying down the safety valves and burning tissue in the furnace. There is a physiological measure of excitement that is good for the individual, for it stirs up the nutritive processes, clears out the cobwebs in the brain, and makes the mind clearer and stronger for it. But excessive and abnormal excitement has burned the youth out of many a body, leaving the possessor an old man at forty. Stock exchanges are notorious fields of shattered nerves and softened brains. Political campaigns may bring party triumphs, but yearly they bring enormous order drains upon vitality to thousands. As strange as it may seem, few indeed in the active whirl of life have that poise of nerve force, that discipline of the vital functions, which will render them at ease when the day's business shall have been completed; and it is such only that are capable of taking rest to the best advantage or experiencing natural sleep.

How many there are that take their business to bed with them, and when sleep comes it is laborious and fatiguing, more or less disturbed by mutterings, dreams, and restlessness. An overstimulated nervous individual can only with difficulty relax, and let go the things for the time being that have over animated them during the day, and thus find perfect repose; but instead they mechanically disobey all the laws of nature in sleep, simple as they are. They are so blinded by immediate personal interests that the habit of not resting when sleeping has grown to such an extent that to return to natural sleep takes thought and study.

Few who pretend to rest give up entirely to the bed, a dead weight, letting the bed hold them instead of trying to hold themselves on the bed. Watch closely and you will usually see the muscles are tense, if not all over, so nearly that it is fatiguing; the spine seems to be a central point of tension. It touches the bed at each end, it is true, but only in its entirety as far as the individual will permit. The knees are usually drawn up, the muscles of the legs tense, the hands and arms contracted, often clasped above the head, the fingers clinched, either holding the pillow or themselves; the head, instead of letting the pillow have its whole weight, holds itself on the pillow, the throat muscles often contracted, or the muscles of the face drawn one way or another. This may seem exaggerated when it is sleep that we are talking about, but it is indeed too true.

How many poor sleepers are more fatigued when they get up than when they go to bed, and yet natural sleep is "nature's sweet restorer," and always brings perfect recuperation to fit one for the coming day's duties. "If I could only stop myself from thinking," is a complaint often heard, but no one can reason himself or herself out of the habit. Even the knowledge that nothing is gained by it, and that it is a drain on the system, often adds to the difficulty and renders the habit more difficult to abandon. But you say, I must get control, reason must assert herself over every bodily function. Try it. You will find that the strain and nervous tension in trying but add to the difficulty. To use a homely phrase, "It is less difficult to jump out of your boots than out of yourself." If you cannot stop thinking, do not try. Let the thoughts stream ahead if they will, only use enough will power to relax all the muscles, make yourself as heavy as possible in bed, and, while the attention of the mind is drawn to the letting-go process of the muscles, the imps of thought find less to do in the brain, because the mind is absorbed in a better work, and soon the senseless thinking will stop.

Five minutes of complete rest are worth more than an hour of common resting. There is no better way of learning to overcome perverseness in sleeping than to study with care the sleep of a healthy little child. Having gained the necessary freedom to give perfect repose, the dropping of all thought and care can be made an easy task. To take the regular process, first let go the muscles, which will enable one more easily to drop disturbing thoughts or refuse without resistance to admit the thoughts, and freedom from care for the time will follow. Take plenty of time for repose, and above all things, court a clear conscience before resting; the latter adds much to tranquil sleep.—*W. H. Maxon, in the Pacific Health Journal.*

